# Associations of employment sector and occupational exposures with full and part-time sickness absence: random and fixed effects analyses on panel data

**DOI:** 10.5271/sjweh.4003

**Published:** 2022-02-25

**Authors:** Elli Hartikainen, Svetlana Solovieva, Eira Viikari-Juntura, Taina Leinonen

**Affiliations:** 1 The Finnish Institute of Occupational Health, P.O. Box 40, 00032 TYÖTERVEYSLAITOS, Finland

**Keywords:** absenteeism, confounding, graded return to work, individual characteristics, longitudinal study, random effect, sick leave, sickness benefit, working condition

## Abstract

**Objective:**

We aimed to investigate the influence of unobserved individual characteristics in explaining the effects of work-related factors on full (fSA) and part-time sickness absence (pSA).

**Methods:**

We used register-based panel data for the period 2005–2016 on a 70% random sample of the Finnish working-age population. The relationships between employment sector and occupational exposures (% exposed to physically heavy work and job control score based on job exposure matrices) and the annual onset of fSA and pSA were investigated among men and women. First, random effects (RE) models were applied controlling for observed sociodemographic factors and then fixed effects (FE) models that examine within-individual changes over time and thereby further account for unobserved time-invariant individual characteristics.

**Results:**

In the RE analyses, public employment sector, physically heavy work and lower job control each increased the use of fSA and pSA among both genders. When unobserved individual characteristics were controlled for with the FE models, the effects on fSA attenuated. For pSA, the effects of employment sector and physical heaviness of work among women even reversed. The effect of lower job control on pSA remained especially among women.

**Conclusions:**

The role of individuals’ unobserved characteristics in explaining the effects of work-related factors on SA should not be neglected. The effects of work-related factors are likely to be overestimated when using traditional approaches that do not account for unobserved confounding, ie, selection of individuals with a high likelihood of SA into particular work environments.

Sickness absence (SA) is known to be influenced by various individual characteristics, being more common, eg, among women than men (1, 2) and those with poorer health or chronic disease (3–6). Furthermore, the contribution of work-related factors is strong. SA rates have been found to be higher among those who work in manual occupations (7–12), are employed in the public sector (9, 13–15), participate in health and social work (11, 14, 16), and are exposed to unfavorable physical and psychosocial working conditions, such as physically heavy work and low job control (17–22).

Previous literature has not been able to fully explain why certain groups of employees have higher rates of SA than others, eg, the differences are especially large between employment sectors, SA rates being clearly higher in the public sector. This difference has been shown to remain even when many important factors that are known to affect SA, such as gender, age, occupational class, education, and income, are being controlled for. The difference in SA has shown to be clear even when comparing the employment sectors within same field of industry, such as health and social work (23).

Partial sickness allowance is a benefit that enables employees with work disability to be absent from work for rest, treatment or rehabilitation and still remain working for a proportion of the time. Partial sickness benefits and graded return-to-work have been increasingly used in the Nordic countries (24) and continental European countries such as Germany and Austria (25) to support part-time work participation during sickness and to facilitate faster full work resumption. There are yet only a few studies concerning the use of partial sickness benefits in different groups of employees (26, 27). In Finland, it has been shown that among employees using any sickness benefits, the use of the partial compared to full benefit was more common in the private than public sector. Occupational exposures, including physical heaviness of work and job control, instead were detected to have only faint and inconsistent associations with the use of partial sickness benefits (26). The association of work-related factors with work ability and the overall need for SA therefore appear to be a different matter from their association with being able to take the leave as part-time. The effects of work-related factors on full and part-time SA (fSA and pSA, respectively) may thus be very different.

In addition to the potential causal mechanisms between work-related factors and SA, the associations may also be affected by selection, ie, individuals with characteristics associated with a high likelihood of fSA or pSA ending up in particular types of jobs. Most previous studies, however, have not been able to adequately control for such confounding, as many individual-level characteristics that are known to affect SA, eg, personality factors such as neuroticism (28, 29), remain unobserved.

One way of accounting for unobserved confounding is to examine the associations of work-related factors with SA among individuals moving between different work environments. By examining within-individual changes in work-related factors over time, the individuals can serve as their own controls accounting for all of their unobserved time-invariant characteristics.

In this article, we apply random effects (RE) and fixed effects (FE) analyses using register-based panel data from Finland to study the relationship between work-related factors, including employment sector and occupational exposures, with the onset of fSA and pSA. The RE model uses both between- and within-individual variations, whereas the individual FE model uses only within-individual variation over time, ie, examines individuals’ movements between employment sectors or occupations with different exposures. To investigate unobserved confounding, we assessed how the examined relationships differed when not only observed time-variant and -invariant individual characteristics were controlled for, as was done in the RE model, but also unobserved time-invariant individual characteristics were controlled for by using the FE model. As far as we are aware, this type of approach has not been used before to investigate the associations of employment sector and occupational exposures with SA. However, Melsom & Mastekaasa (30) applied a similar methodological approach using Norwegian register data to study the association between occupational gender composition and SA, indicating that the higher number of SA days in female-dominated occupations was fully explained by selection based on individual characteristics. Longitudinal individual-level register data enable the use of this method, since it requires panel data with a large number of subjects followed up over several repeated measurements.

In this study, our aim was therefore to discover whether unobserved time-invariant individual characteristics explain the associations of work-related factors with the onset of fSA and pSA, ie, whether the differences by employment sector and occupational exposures are related to selection of individuals into working in these circumstances, in addition to potential system-based or work-driven reasons.

## Methods

### Study design

We used a large, 70% random sample of the working-age population living in Finland on the last day of year 2004. The register-based data included individual-level information on episodes of compensated SA and national pensions obtained from the Finnish Social Insurance Institution, episodes of employment and earnings-related pensions from the Finnish Centre for Pensions, as well as on sociodemographic and employment-related factors from the FOLK data of Statistics Finland. In the analyses of fSA, the study period covered 2005–2016. pSA has only been available since 2007 and the study period therefore started two years later.

For this study, we included wage earners who (i) turned 30–62 years during a study year, (ii) did not receive any pension or vocational rehabilitation allowance, and (iii) were employed in the private or public sector on the first day of that year. It was also required that the individual was not employed in both sectors during a study year and had information on work-related factors available. We only included individuals who, at the beginning of the study year, were not already on either fSA or pSA, depending on the analysis. The individual nevertheless could have had SA in the preceding years. The criteria for being included in the study population were applied separately to each study year. An individual could thus be excluded in one year and included in others. Because of the panel design, however, it was required that a person was eligible in at least two study years to be included in the study population [fSA analyses: 2–12 (mean 7.8) years; pSA analyses: 2–10 (mean 6.9) years].

### Full- and part-time sickness absence

In Finland, the Social Insurance Institution of Finland compensates permanent residents for SA after a waiting period of ten working days (including Saturday) that is typically paid by the employer (31). The outcome of this study was therefore based on longer-term SA that lasted around two calendar weeks or more. We examined whether a study person had a new onset of fSA or pSA during each study year. pSA is a voluntary option for persons who are eligible for fSA if medical assessment shows they can work without harm to their health and their employer can arrange part-time work. Between 2007 and 2009, pSA could only start after 60 compensated fSA days. Since 2010, it has been possible to have pSA immediately from the first compensated day, but it has still typically been preceded by a compensated fSA episode. A person can also transition into fSA from pSA.

Because of the different inclusion criteria and the different lengths of the study periods, the final study populations of the fSA and pSA analyses were not fully the same, although very similar (table 1). The fSA analyses were based on 1 460 086 individuals with a total of 11 383 552 person years, ie, observation years, in the period 2005–2016, while the pSA analyses included 1 379 446 individuals with 9 581 811 person years in the period 2007–2016. This resulted in around 900 000 to almost 1 000 000 individuals per study year (supplementary material, www.sjweh.fi/article/4003, tables S1 and S2).

**Table 1:** Distribution of the observation years in the period 2005–2016 for the full sickness absence (fSA) data and in the period 2007–2016 for part-time sickness absence (pSA) data by sociodemographic and work-related factors.

	fSA data		pSA data	
	
	N	%	N	%
Gender				
Men	5 504 904	48.4	4 616 381	48.2
Women	5 878 648	51.6	4 965 430	51.8
Age group (years)			
30–34	1 666 777	14.6	1 408 040	14.7
35–39	1 726 799	15.2	1 437 871	15.0
40–44	1 812 875	15.9	1 493 318	15.6
45–49	1 920 712	16.9	1 608 003	16.8
50–54	1 905 571	16.7	1 602 398	16.7
55–59	1 710 442	15.1	1 457 141	15.2
60–62	640 396	5.6	575 040	6.00
Living arrangements			
Alone	1 934 472	17.0	1 643 220	17.1
With partner only	2 894 522	25.4	2 451 499	25.6
With partner and at least one child	5 392 628	47.4	4 512 641	47.1
Lone parent with at least one child	676 988	5.9	571 380	6.0
Other	484 942	4.3	403 071	4.2
Income (€/year) ^a^			
≤20 000	1 127 154	9.9	852 162	8.9
≤40 000	6 555 150	57.6	5 403 953	56.4
≤60 000	2 568 352	22.5	2 300 849	24.0
>60 000	1 132 896	10.0	1 024 847	10.7
Education			
Primary	1 436 499	12.6	1 135 803	11.9
Secondary	4 801 156	42.2	4 076 564	42.5
Tertiary	5 145 897	45.2	4 369 444	45.6
Region			
Uusimaa (capital region)	3 695 813	32.5	3 107 907	32.4
Southern	1 434 103	12.6	1 203 397	12.6
Western	3 913 759	34.4	3 297 488	34.4
Eastern	1 069 140	9.4	898 034	9.4
Northern	1 270 737	11.1	1 074 985	11.2
Employment sector			
Private	7 552 771	66.4	6 370 098	66.5
Public	3 830 781	33.6	3 211 713	33.5
Physically heavy work ^a^			
<40% exposed	8 073 976	70.9	6 846 902	71.5
≥40% exposed	3 309 576	29.1	2 734 909	28.5
Job control score ^a^			
>Median (high)	4 776 325	42.0	4 077 168	42.6
≤Median (low)	6 607 227	58.0	5 504 643	57.4
Total	11 383 552	100	9 581 811	100.0

a Income, physically heavy work, and job control score have been categorized for descriptive purposes.

### Work-related factors

Employment sector during the study year was classified into private and public sector based on the sector of the pension-insured employment (32).

Occupational exposures were estimated by linking information from job exposure matrices (JEM) that were developed earlier in a large population survey- and interview-study and have been described in more detail elsewhere (33, 34). We chose to examine physical heaviness of work as a general measure encompassing various specific physical exposures. With respect to psychosocial exposures, we chose to examine job control, for which the JEM had better validity than, eg, for job demands. The JEM provide information on the proportion reporting physically heavy work (range 0–100%) and the mean job control score (range 1–5, with higher value indicating higher control based on the Karasek concept) (35) in men and women holding a specific occupational title.

Occupational exposures were used as continuous variables in the regression analyses. For descriptive purposes they were dichotomized using 40% exposed to physically heavy work and gender-specific median job control score as cut-off points. The information is available for most occupations, almost 400 in total. The previously developed JEM information was linked to occupational titles of the individuals in the present register data, using a classification by Statistics Finland (36), based on the International Standard Classification of Occupations (ISCO-88). Information on occupational titles was available in the register data at the end of each calendar year since 2004. We used the most recent recorded information before each study year.

### Statistical methods

The relationships of the three work-related factors (employment sector, physical heaviness of work and job control) and the two different outcomes (fSA and pSA) were analysed using panel regression with RE and FE analysis. The RE regression model was considered as our baseline model since it uses all observed information (ie, both between and within variations, accounting for clustering of observations within individuals), whereas the individual FE regression uses only within-individual observations that change over time. As it is, FE regression controls for all observed time-variant and -invariant as well as unobserved time-invariant individual characteristics in the measurement period (37, 38). By comparing the FE results with the ones from the RE regression model, which only controls for the observed time-variant and -invariant individual characteristics, the role of unobserved time-invariant characteristics in the associations of employment sector and occupational exposures with fSA and pSA could be assessed.

We used linear probability models for analysing the binary outcome instead of logistic regression models since the logistic regression FE models do not include in its analytic sample study subjects whose outcome remains unchanged over time (ie, those whose response variable is always coded as 0 or as 1). Using the latter model would make the comparison of results from the RE and FE models to assess the role of unobserved time-invariant individual characteristics problematic. In linear probability models, the regression coefficients are on an absolute scale, which can be interpreted as the absolute difference in the share of the occurrence of the outcome in terms of the explanatory variable. We present the results as percentage point differences.

The RE and FE models were estimated for the onset of both fSA and pSA so that employment sector and occupational exposures were considered as explanatory variables. The work-related factors were mutually controlled for and additionally controlled for other background variables that are known to be associated with SA: gender, age (age-groups between five years), living arrangements (alone, with partner only, with partner and ≥1 child, lone parent with ≥1 child and other), inflation-corrected taxable total income earned by the individual (continuous variable), educational level (with three classes), region of residence and calendar year. The analyses were performed also stratifying by gender.

Since the FE analyses only uses information on the study subjects whose work-related factors changed over the study period, we present sensitivity analyses in which we performed the RE analyses among the subpopulations of “changers”. By doing so we could assess whether the associations of work-related factors with fSA and pSA differed between the “changers” and the general population. In addition, we tested the assumption of linear associations of physical heaviness of work and job control score with the outcomes by repeating the main analyses using categorised exposure variables.

All analyses were performed using STATA15 (StataCorp, College Station, TX, USA).

## Results

Sociodemographic and work-related factors were very similar between the observations included in the fSA and pSA analyses (table 1). In both the fSA and pSA datasets, working in the private sector was clearly more common than working in the public sector, the former occurring in 66% of the observation years. Overall, physically heavy work was common (≥40% in the occupation exposed) in approximately 29% of the observation years. The prevalence of job control below median was 57% of the observation years.

The proportions of onset of both fSA and pSA were higher among those who worked in the public sector or had physically heavy work or low job control (table 2). Overall, an onset of fSA and pSA occurred in 12.5% and 0.44% of the observation years, respectively. There was, nevertheless, variation in these proportions over the study period. The annual proportions of onset of fSA decreased (supplementary table S1) whereas the annual proportions of onset of pSA largely increased (supplementary table 2) in all variable groups. The proportions of onset of both fSA and pSA also differed between genders (table 2) being clearly greater among women than among men.

**Table 2:** Proportion of observation years with an onset of full (fSA) and part-time (pSA) sickness absence by sociodemographic and work-related factors.

	% of onset of fSA			% of onset of pSA		
	
	Men	Women	All	Men	Women	All
Age group (years)						
30–34	6.9	10.6	9.2	0.14	0.37	0.25
35–39	8.0	12.3	10.1	0.19	0.47	0.33
40–44	9.2	13.3	11.3	0.19	0.55	0.38
45–49	10.4	14.8	12.7	0.22	0.63	0.43
50–54	12.0	16.6	14.5	0.28	0.78	0.55
55–59	13.6	17.8	15.9	0.33	0.87	0.63
60–62	13.4	17.3	15.6	0.29	0.68	0.51
Living arrangements						
Alone	10.5	15.6	12.9	0.26	0.74	0.49
With partner only	11.3	16.5	14.1	0.26	0.74	0.51
With partner and at least one child	9.3	13.1	11.2	0.20	0.51	0.36
Lone parent with at least one child	12.1	16.8	16.0	0.28	0.71	0.64
Other	9.8	14.5	11.4	0.22	0.62	0.36
Income (€/year) ^a^						
≤20 000	7.9	13.2	11.7	0.08	0.24	0.20
≤40 000	12.1	16.4	14.7	0.27	0.75	0.56
≤60 000	9.5	11.1	10.1	0.23	0.53	0.34
>60 000	5.7	8.6	6.4	0.14	0.42	0.21
Education						
Primary	14.0	19.4	16.3	0.27	0.66	0.43
Secondary	12.0	17.9	14.7	0.26	0.78	0.50
Tertiary	6.4	11.5	9.4	0.17	0.51	0.37
Region						
Uusimaa (capital region)	8.3	12.6	10.5	0.22	0.55	0.39
Southern	11.6	15.6	13.6	0.21	0.59	0.40
Western	10.6	15.5	13.1	0.25	0.68	0.47
Eastern	11.9	16.3	14.2	0.23	0.66	0.45
Northern	10.9	16.6	13.8	0.21	0.76	0.49
Employment sector						
Private	9.7	12.8	10.9	0.23	0.58	0.37
Public	12.0	16.8	15.6	0.23	0.68	0.57
Physically heavy work ^a^						
<40% exposed	8.1	13.1	10.9	0.21	0.57	0.41
≥40% exposed	13.7	20.6	16.4	0.26	0.85	0.49
Job control score ^a^						
>median (high)	7.3	13.0	10.2	0.18	0.53	0.36
≤median (low)	12.2	16.0	14.2	0.27	0.70	0.49
Total	10.1	14.8	12.5	0.23	0.63	0.44
Number of events	556 551	867 305	1 423 856	10 490	31 255	41 745
N	5 504 904	5 878 648	11 383 552	4 616 381	4 965 430	9 581 811

a Income, physically heavy work, and job control score have been categorized for descriptive purposes.

In the dataset used for the fSA analyses, 7.3% (N=105 894) of the individuals changed their employment sector at least once during the study period. In the pSA data, the amount was 6.2% (N=85 189). Both physical heaviness of work and job control score changed for around 60% of employees (physical heaviness: 60.0%, N=875 699 in fSA data; 57.3%, N=790 326 in pSA data; job control score: 62.0%, N= 906 466 in fSA data; 59.8%, N=824 423 in pSA data).

Results from the regression analyses on the associations of employment sector and the two examined occupational exposures with fSA and pSA, adjusted for sociodemographic factors and mutually for the work-related factors, are presented in tables 3 and 4, respectively, addressed below. The supplementary material provides corresponding results, but with adjustment only for age and gender (supplementary tables S3 and S4) and adjustment for all sociodemographic factors but without mutual adjustment for the work-related factors (supplementary tables S5 and S6). The comparison of the results from the RE and FE models was largely similar regardless of the used adjustments.

**Table 3:** Results of random (RE) and fixed (FE) effects regression analyses on the associations of work-related factors with full sickness absence (fSA) among men and women, percentage point differences

	fSA			

	RE ^a^		FE ^a^	
	
	Estimate	95% CI	Estimate	95% CI
Men				
Public sector (vs. private)	3.279	3.175–3.383	-0.182	-0.465–0.101
Physically heavy work ^b^	0.752	0.721–0.784	0.124	0.073–0.176
Job control ^c^	2.581	2.490–2.672	0.482	0.363–0.600
Women				
Public sector (vs. private)	4.068	3.979–4.157	0.726	0.490–0.962
Physically heavy work ^b^	1.870	1.827–1.913	0.278	0.208–0.348
Job control ^c^	1.184	1.075–1.292	0.323	0.164–0.481
All				
Public sector (vs. private)	3.890	3.825–3.955	0.392	0.209–0.576
Physically heavy work ^b^	1.224	1.200–1.249	0.186	0.144–0.228
Job control ^c^	1.890	1.820–1.960	0.370	0.274–0.465

a Models controlled for age, gender, educational level, income, living arrangements, year, region and mutually for the work-related factors.

b 20 percentage point increase in the % exposed.

c One unit decrease in the score.

**Table 4:** Results of random (RE) and fixed effects (FE) regression analyses on the associations of work-related factors with part-time sickness absence (pSA) among men and women, percentage point differences.

	pSA			

	RE ^a^		FE ^a^	
	
	Estimate	95% CI	Estimate	95% CI
Men				
Public sector (vs. private)	0.030	0.018–0.043	-0.041	-0.062– -0.019
Physically heavy work ^b^	0.002	-0.002–0.006	0.002	-0.007–0.011
Job control ^c^	0.126	0.111–0.140	0.036	0.015–0.057
Women				
Public sector (vs. private)	0.106	0.089–0.122	-0.178	-0.233– -0.122
Physically heavy work ^b^	0.083	0.074–0.091	-0.061	-0.080– -0.042
Job control ^c^	0.138	0.116–0.160	0.115	0.074–0.156
All				
Public sector (vs. private)	0.096	0.084–0.107	-0.127	-0.168– -0.086
Physically heavy work ^b^	0.035	0.031–0.039	-0.019	-0.028– -0.009
Job control ^c^	0.150	0.137–0.163	0.099	0.078–0.120

a Models controlled for age, gender, educational level, income, living arrangements, year, region and mutually for the work-related factors.

b 20 percentage point increase in the % exposed.

c One unit decrease in the score.

### The relation of employment sector with full and part-time sickness absence

Using RE panel regression that uses all observed information (ie, both between and within variations) and controlling for background variables, fSA was more common in the public sector compared to the private sector (3.890, table 3) among the overall study population. The coefficient can be interpreted as the absolute percentage point difference in the share of fSA for study subjects working in the public compared to private sector. With pSA, the difference was in the same direction, being approximately 0.10 percentage points (table 4). The relative magnitude of these differences can be addressed in relation to the average shares of fSA and pSA in the data, 12.5% and 0.44%, respectively. The effect of employment sector was greater among women than men in both the fSA and pSA analyses, which is understandable, since the average shares of these outcomes in the data were much higher among women (table 2).

In the FE model, when all observed and unobserved time-invariant individual characteristics were controlled for, the percentage point difference between the employment sectors among the overall study population was clearly smaller (0.392) than in the RE results. This FE coefficient indicates that the share of fSA was around 0.4 percentage points larger in the observation years during which a study person was employed in the public sector compared to the years in private sector employment. Using FE analysis within gender groups, this relation was found only among women. In turn, among the overall study population, the share of pSA was 0.13 percentage points smaller during employment in the public than in the private sector, being contrary to the results from RE model, where the relation was the reverse. This relation was clearly greater among women than men.

### The relation of occupational exposures with full and part-time sickness absence

According to our RE analyses, a 20 percentage point increase in the proportion exposed to physically heavy work increased the share of fSA by 1.22 percentage points (table 3) and pSA by 0.04 percentage points (table 4). Again, the effect on fSA was larger among women, whereas the effect on pSA was found only among women. In the FE analysis performed for the overall study population, the effect of physically heavy work on fSA largely attenuated, ie, to 0.19 percentage points. This effect was still larger among women than men. A 20 percentage point increase in physically heavy work had a decreasing, yet faint (approximately 0.02 percentage points), effect on pSA. The association was contrary to the RE result, but again only found among women.

In the RE analyses, a one unit decrease in the job control score increased the share of both fSA and pSA with 1.89 and 0.15 percentage points, respectively. With fSA the effect was clearly larger among men than women, whereas with pSA the differences between the genders were minor. In the FE analyses, the effect of decreasing job control score on both fSA and pSA attenuated, being still positive, yet fainter compared to the RE model (ie, being 0.37 percentage points with fSA and 0.10 percentage points with pSA). The effect on fSA was still larger among men than women, whereas the effect on pSA attenuated less among women than men, being now larger among the former.

### Sensitivity analyses

The associations of physical heaviness of work and job control with fSA and pSA in the RE analyses including the subpopulation whose work-related factors changed over the study period (supplementary table S7) were in line with those in the main RE analyses (tables 3 and 4). The effects of public sector employment with fSA were smaller in the RE analyses including the “changers” than in the main RE analyses, but nevertheless explained further in the FE analyses. The effects of employment sector on pSA reversed in the RE analyses including the “changers” compared to the main RE analyses, but among women became even more reversed in the FE analysis.

Supplementary tables S8 and S9 showed linearity in the associations of categories of physical heaviness of work and job control score with fSA and pSA in our baseline RE analyses.

## Discussion

The observed associations between work-related factors and SA may be related to factors that are characteristic to the individual. To address this, we used large register-based panel data and applied RE models controlling for sociodemographic factors, both changing and unchanging over time. FE models were then used to further account for unobserved individual characteristics that are unchanged over time.

Based on the RE models, our findings showed that the onset of both fSA and pSA was more common in the public than the private employment sector for both genders. Physically heavy work and lower job control increased the likelihood of both fSA and pSA, although the effect of physically heavy work on pSA was found only among women. It was also noticeable that in the fSA analyses, the effect of physically heavy work was greater among women, whereas lower job control had a greater effect among men.

After additionally adjusting for unobserved individual characteristics that were unchanged over time (using the FE models), the associations largely attenuated for fSA. Among men fSA no longer varied by employment sector. For pSA some of the associations even became reverse, the onset of pSA being more common in the private than public sector specially among women. Furthermore, among women the onset of pSA became more common as the likelihood of being exposed to physically heavy work decreased. Finally, the effect of lower job control on pSA attenuated specially among men.

Our results on fSA, based on the traditional approach adjusting for observed factors (RE model), were parallel with previous findings on the effects of public sector employment (9, 13–15), physically heavy work (17–19) and low job control (17, 20–22) on SA. Factors associated with pSA have been investigated less, the focus being eg, on the use of the partial compared to the full benefit (26, 27).

Our novel results based on the comparison of the FE models with the RE models suggest that the typically observed associations of employment sector and occupational exposures with SA may to a large extent be explained by unobserved individual characteristics. Underlying personal factors, eg, certain personality traits appear to influence both selection into particular work environments (39–42) and having a high likelihood of SA (28, 29, 43). In addition, work attitudes and values have been found to influence selection into particular employment sectors (44–46) and these factors may also be associated with the use of SA. The method applied and our findings, together with previous ones (30), underline the importance of considering how unmeasured confounding by individual-level factors can affect the results. Register data, in particular, have limited information on important personal factors, and its use may therefore lead to an overestimation of the effect of work-related factors on SA.

The role of unobserved individual characteristics was generally larger in pSA compared with fSA. This finding can be understood by the voluntary choice of pSA instead of fSA in Finland. The remaining impact of work-related factors may relate to differences between work environments in the feasibility and benefit of the use of pSA (27). Our finding on the remained importance of lower job control in pSA, particularly among women, could be interpreted as the use of pSA being an employee’s way to increase the time control in the job.

The more common use of pSA in the private than the public sector after controlling for unobserved individual factors suggests that the private sector might be more capable of making use of part-time work during pSA. Providing the possibility for pSA would also be an economic incentive for the employer, who will in most cases pay full salary to the employee at least during the first months of fSA.

After controlling for unobserved individual factors, the use of pSA was common among women with physically less heavy jobs. Reducing the heaviness of exposure by work modifications might enhance the use of pSA. However, these modifications may not be easily implemented in physically heavy jobs, which could explain the reverse association between physically heavy work and the use of pSA.

The use of pSA and graded return to work have been found to increase work participation (47–56) ie, promote the recovery and full return to work. In addition to the financial implications, reduced work participation at working age has adverse individual- and population-level effects on health and wellbeing (57), and pSA as an alternative for fSA is an effective way to decrease those effects. Future studies should investigate further why certain groups of employees are better able to return to part-time work while being sick.

Our study has several important strengths. Our findings were drawn from rich data of a very large and nationally representative register-based sample, which included sufficient within-individual changes in work-related factors that enabled us to apply the individual FE model alongside of the RE model. Also, with the register-based data there is no problem of non-response or loss to follow-up.

In our analyses, we estimated occupational exposures using JEMs, which offer information of exposures on the occupational level. Linking the JEMs to occupational titles in the register data enabled us to assess work-related factors on which individual-level information was unavailable.

We also acknowledge that there are some limitations. First of all, the FE models do not control for time-varying individual factors such as changes over time in life situations that were unmeasured in our study but could affect both working-career choices and having SA. As it is, the role of these factors as confounders cannot be estimated.

Secondly, the FE models do not use information from study subjects whose work-related factors remained the same over the study period. The FE model provides subject-specific estimates for the subpopulation that experienced a change in work-related factors, whereas RE models are based on the effects of the total population average. Since the results derived from the FE analysis are based on a subgroup that is prone to move between employment sectors or occupations and might therefore differ from the overall working population, we performed sensitivity analyses limiting the RE analyses to the subpopulations whose work-related factors changed over the study period. These analyses indicated that the associations of physically heavy work and job control with the SA outcomes among the “changers”, covering around 60% of the study population, were in line with those in the total study population. Only 6–7% of the study population changed their employment sector, this subpopulation therefore being more selected and within-individual changes weighing more in the analyses. The effects of employment sector were smaller for fSA and reversed for pSA among the “changers” compared to the total study population but – with the exception of pSA for men – were explained further in the FE analyses. Both public sector employment and the use of pSA are relatively uncommon among men, the group therefore being very selected. Overall, our findings indicate that at least among employees who have relatively high mobility in the labor market, work-related factors appear to affect SA to a limited extent.

It is also noticeable that since the JEM are based on aggregated occupation-level information, they do not capture variation in the occupational exposures within an occupation. We therefore observed changes in the exposures resulting from a change in the individual’s occupation, but not from eg, work modifications or changes in work tasks made within one’s current occupation. In general, the effects of exposures measured by JEM have a tendency towards null association. Taking into consideration the factors mentioned above, the influences of occupational exposures on SA are likely to have been somewhat underestimated in both approaches of our study.

### Concluding remarks

The findings of the current study suggest that the role of individuals’ unobserved characteristics in explaining the effect of work-related factors – employment sector, physical heaviness of work and job control – on SA should not be neglected. The effects of work-related factors are likely to be overestimated when using traditional approaches that do not account for unobserved confounding, ie, selection of individuals with a high likelihood of SA into particular work environments.

## Supplementary material

Supplementary material
