# Supplementary material for: Associations of employment sector and occupational exposures with full and part-time sickness absence: random and fixed effects analyses on panel data
Source: Scand J Work Environ Health. 2022 Feb 25;48(2):148–57. doi: 10.5271/sjweh.4003 (PMC9045233; doi:10.5271/sjweh.4003)
Supplement: Supplementary material [file SJWEH-48-148-S001.pdf]

Associations of employment sector and occupational exposures with full and part-time sickness absence: random and fixed effects analyses on panel data[1]

by Elli Hartikainen, MSc,[2] Svetlana Solovieva, PhD, Eira Viikari-Juntura, MD, PhD, Taina Leinonen, PhD

1. Supplementary material
2. Correspondence to: Elli Hartikainen, The Finnish Institute of Occupational Health, P.O. Box 40, 00032 TYÖTERVEYSLAITOS, Finland. [E-mail: elli.hartikainen@ttl.fi]

Supplementary table S1. Annual proportions of onset of full sickness absence by sociodemographic and work-related factors

	2005	2006	2007	2008	2009	2010	2011	2012	2013	2014	2015	2016
**Gender**												
Men	11.0	11.4	11.3	11.0	10.0	10.2	10.2	9.7	9.5	9.3	8.8	8.8
Women	16.0	16.3	15.8	15.6	14.8	15.0	15.0	14.0	14.0	13.8	13.4	13.4
**Age group (years)**												
30–34	10.3	10.1	9.8	9.7	9.4	9.3	9.3	8.8	8.6	8.5	8.1	8.3
35–39	11.4	11.2	10.9	10.7	9.9	10.2	10.2	9.6	9.4	9.3	9.0	8.8
40–44	12.8	12.5	12.1	11.9	11.2	11.4	11.4	10.8	10.7	10.5	9.9	9.9
45–49	14.3	14.3	14.0	13.7	12.6	12.8	12.9	12.1	12.0	11.5	11.1	11.1
50–54	16.0	16.3	15.9	15.5	14.6	14.7	15.8	13.9	13.5	13.2	12.6	12.4
55–59	16.3	17.8	17.5	17.3	16.1	16.0	16.1	15.1	15.1	14.9	14.3	14.3
60–62	14.8	16.8	16.6	16.6	15.7	16.0	15.9	14.7	15.3	15.0	14.8	14.9
**Living arrangements**												
Alone	13.6	14.1	14.1	13.9	12.8	13.1	13.1	12.3	12.2	12.0	11.5	11.7
With partner only	14.9	15.3	15.3	14.9	14.1	14.3	14.3	13.5	13.4	13.3	12.5	12.8
With partner and at least one child	12.6	12.2	12.2	12.0	11.2	11.3	11.3	10.6	10.5	10.3	9.9	9.8
Lone parent with at least one child	17.4	16.8	16.8	16.8	16.1	16.3	16.2	15.4	15.4	15.1	14.8	14.7
Other	12.0	12.1	12.1	12.2	11.0	11.4	11.6	11.0	11.0	11.0	10.5	10.5
**Income (€/year)**												
<=20 000	13.7	13.4	12.9	12.6	11.6	11.4	11.1	10.6	10.0	9.5	9.1	9.1

<=40 000	15.1	15.6	15.4	15.3	14.5	14.7	14.9	14.2	14.2	13.9	13.5	13.8
<=60 000	9.7	10.0	10.2	10.4	10.1	10.2	10.5	10.0	10.1	10.2	9.8	9.8
>60 000	6.0	6.3	6.3	6.7	6.3	6.4	6.7	6.5	6.6	6.5	6.4	6.3
**Education**												
Primary	17.0	17.9	17.6	17.1	16.0	16.5	16.4	15.5	15.2	15.2	14.3	14.4
Secondary	15.8	16.1	15.8	15.6	14.6	14.9	15.0	14.2	14.1	13.8	13.4	13.5
Tertiary	10.1	10.1	9.9	9.9	9.5	9.5	9.5	9.0	9.0	8.9	8.6	8.7
**Region**												
Uusimaa (capital region)	11.6	11.8	11.4	11.2	10.7	10.8	10.7	10.0	9.9	9.7	9.3	9.2
Southern	14.4	14.4	14.6	14.6	13.4	13.6	14.1	13.4	13.2	12.9	12.6	12.5
Western	14.3	14.7	14.3	14.1	13.1	13.2	13.3	12.4	12.3	12.0	11.6	11.8
Eastern	15.5	15.8	15.6	15.0	14.2	14.3	14.3	13.6	13.5	13.2	12.5	12.9
Northern	14.5	15.2	14.9	14.6	13.6	14.0	14.0	13.2	13.1	13.1	12.6	12.6
**Employment sector**												
Private	11.9	12.3	12.0	11.8	10.9	11.1	11.1	10.5	10.3	10.1	9.6	9.6
Public	16.6	16.9	16.6	16.5	15.8	15.8	15.9	14.8	14.8	14.6	14.3	14.4
**Physically heavy work**												
<40% exposed	11.6	11.8	11.5	11.4	10.9	10.9	11.2	10.6	10.6	10.4	10.1	10.1
>=40% exposed	17.7	18.2	17.9	17.4	16.1	16.5	16.4	15.6	15.3	15.1	14.4	14.6
**Job control score**												
>median (high)	11.0	11.1	10.9	10.8	10.4	10.4	10.3	9.80	9.8	9.7	9.4	9.4
<=median (low)	15.2	15.6	15.3	15.0	13.9	14.2	14.5	13.6	13.5	13.2	12.7	12.8
Total	13.6	13.9	13.6	13.4	12.5	12.7	12.7	11.9	11.8	11.6	11.2	11.2
Number of events	123 401	133 471	131 186	130 718	122 774	121 431	120 990	114 077	112 566	109 189	103 976	100 077
N	910 002	962 384	967 024	979 410	981 750	959 125	953 607	955 653	950 693	941 252	930 683	891 969

Supplementary table S2. Annual proportions of onset of part-time sickness absence by sociodemographic and work-related factors

	2007	2008	2009	2010	2011	2012	2013	2014	2015	2016
**Gender**										
Men	0.05	0.05	0.06	0.14	0.19	0.23	0.30	0.38	0.43	0.47
Women	0.13	0.15	0.16	0.42	0.54	0.70	0.81	1.00	1.13	1.29
**Age group(years)**										
30–34	0.05	0.05	0.06	0.18	0.21	0.28	0.36	0.40	0.46	0.54
35–39	0.07	0.07	0.08	0.21	0.26	0.37	0.40	0.51	0.63	0.65
40–44	0.08	0.09	0.10	0.26	0.34	0.40	0.53	0.65	0.72	0.80
45–49	0.11	0.12	0.12	0.29	0.39	0.49	0.60	0.70	0.79	0.84
50–54	0.13	0.14	0.15	0.39	0.50	0.61	0.73	0.88	0.93	1.08
55–59	0.13	0.14	0.16	0.40	0.53	0.69	0.78	1.06	1.13	1.24
60–62	0.04	0.08	0.09	0.21	0.31	0.47	0.57	0.73	0.95	1.18
**Living arrangements**										
Alone	0.10	0.11	0.14	0.34	0.42	0.53	0.61	0.80	0.84	0.97
With partner only	0.10	0.12	0.13	0.32	0.42	0.56	0.67	0.84	0.95	1.09
With partner and at least one child	0.08	0.09	0.09	0.23	0.31	0.40	0.48	0.56	0.66	0.73
Lone parent with at least one child	0.17	0.15	0.15	0.42	0.57	0.68	0.84	1.02	1.14	1.33
Other	0.07	0.07	0.08	0.24	0.33	0.36	0.47	0.63	0.70	0.72
**Income (€/year)**										
<=20 000	0.06	0.07	0.09	0.17	0.22	0.30	0.29	0.33	0.36	0.39
<=40 000	0.12	0.13	0.14	0.36	0.48	0.63	0.76	0.96	1.10	1.26
<=60 000	0.05	0.06	0.07	0.19	0.25	0.32	0.42	0.52	0.55	0.66
>60 000	0.05	0.04	0.04	0.14	0.19	0.21	0.24	0.28	0.34	0.36
**Education**										
Primary	0.08	0.12	0.10	0.28	0.42	0.56	0.66	0.83	0.94	1.04
Secondary	0.11	0.11	0.13	0.31	0.41	0.54	0.66	0.82	0.93	1.07
Tertiary	0.09	0.09	0.10	0.26	0.32	0.40	0.46	0.58	0.64	0.73
**Region**										

Uusimaa (capital region)	0.07	0.09	0.09	0.24	0.36	0.42	0.51	0.65	0.71	0.77
Southern	0.10	0.09	0.11	0.26	0.32	0.50	0.55	0.62	0.71	0.86
Western	0.11	0.11	0.12	0.32	0.40	0.50	0.62	0.75	0.85	0.97
Eastern	0.08	0.10	0.12	0.27	0.39	0.49	0.59	0.76	0.83	1.00
Northern	0.11	0.12	0.15	0.35	0.39	0.52	0.58	0.79	0.91	1.03
**Employment sector**										
Private	0.08	0.08	0.08	0.24	0.32	0.41	0.50	0.60	0.67	0.74
Public	0.12	0.14	0.17	0.37	0.47	0.60	0.71	0.92	1.04	1.22
**Physically heavy work**										
<40% exposed	0.09	0.09	0.10	0.27	0.35	0.45	0.52	0.65	0.72	0.83
>=40% exposed	0.10	0.12	0.13	0.32	0.43	0.56	0.71	0.86	1.01	1.12
**Job control score**										
>median (high)	0.08	0.09	0.10	0.24	0.30	0.37	0.44	0.53	0.61	0.70
<=median (low)	0.10	0.11	0.12	0.31	0.43	0.56	0.67	0.85	0.94	1.08
Total	0.09	0.10	0.11	0.29	0.37	0.48	0.57	0.71	0.79	0.90
Number of events	876	1009	1113	2768	3601	4618	5481	6717	7454	8108
N	940 671	989 089	992 757	970 759	965 926	968 103	961 203	952 178	940 496	900 629

Supplementary table S3. Results of random (RE) and fixed effects (FE) regression analyses on the associations of work-related factors with full sickness absence (fSA) among men and women, minimally adjusted models, percentage point differences

	fSA			
	RE[a]		FE[a]	
	**Estimate**	**95% CI**	**Estimate**	**95% CI**
**Men**				
Public sector (vs. private)	2.912	2.808–3.017	-0.173	-0.456–0.109
Physically heavy work (20 percentage point increase in the % exposed)	1.393	1.364–1.421	0.105	0.054–0.157
Job control (one unit decrease in the score)	3.620	3.530–3.709	0.464	0.346–0.582
**Women**				
Public sector (vs. private)	3.808	3.719–3.900	0.747	0.512–0.983
Physically heavy work (20 percentage point increase in the % exposed)	2.424	2.383–2.465	0.251	0.181–0.321
Job control (one unit decrease in the score)	2.458	2.353–2.563	0.244	0.087–0.401
**All**				
Public sector (vs. private)	3.630	3.563–3.700	0.433	0.250–0.616
Physically heavy work (20 percentage point increase in the % exposed)	1.834	1.813–1.858	0.166	0.124–0.208
Job control (one unit decrease in the score)	3.058	2.990–3.126	0.359	0.264–0.455

[a] Models controlled for age and gender (in the model for all)

Supplementary table S4. Results of random (RE) and fixed effects (FE) regression analyses on the associations of work-related factors with part-time sickness absence (pSA) among men and women, minimally adjusted models, percentage point differences

	pSA			
	RE[a]		FE[a]	
	**Estimate**	**95% CI**	**Estimate**	**95% CI**
**Men**				
Public sector (vs. private)	0.018	0.005–0.031	-0.109	-0.159– -0.060
Physically heavy work (20 percentage point increase in the % exposed)	0.005	0.002–0.009	-0.006	-0.015–0.003
Job control (one unit decrease in the score)	0.134	0.121–0.148	0.018	-0.003–0.039
**Women**				
Public sector (vs. private)	0.102	0.085–0.118	-0.112	-0.167– -0.056
Physically heavy work (20 percentage point increase in the % exposed)	0.092	0.084–0.101	-0.079	-0.098– -0.060
Job control (one unit decrease in the score)	0.177	0.155–0.198	0.129	0.088–0.170
**All**				
Public sector (vs. private)	0.085	0.074–0.096	-0.086	-0.127– -0.046
Physically heavy work (20 percentage point increase in the % exposed)	0.041	0.037–0.045	-0.032	-0.042– -0.023
Job control (one unit decrease in the score)	0.168	0.156–0.181	0.086	0.065–0.107

[a] Models controlled for age and gender (in the model for all)

Supplementary table S5. Results of random (RE) and fixed effects (FE) regression analyses on the associations of work-related factors with full sickness absence (fSA) among men and women without mutual adjustment of work-related factors, percentage point differences

	fSA			
	RE[a]		FE[a]	
	**Estimate**	**95% CI**	**Estimate**	**95% CI**
**Men**				
Public sector (vs. private)	2.769	2.666–2.873	-0.232	-0.515–0.050
Physically heavy work (20 percentage point increase in the % exposed)	0.877	0.846–0.908	0.157	0.106–0.208
Job control (one unit decrease in the score)	2.714	2.626–2.802	0.534	0.417–0.651
**Women**				
Public sector (vs. private)	4.013	3.927–4.099	0.705	0.469–0.941
Physically heavy work (20 percentage point increase in the % exposed)	2.074	2.032–2.116	0.310	0.242–0.378
Job control (one unit decrease in the score)	1.284	1.184–1.385	0.450	0.300–0.604
**All**				
Public sector (vs. private)	3.540	3.474–3.607	0.359	0.176–0.543
Physically heavy work (20 percentage point increase in the % exposed)	1.381	1.356–1.406	0.215	0.173–0.256
Job control (one unit decrease in the score)	1.945	1.878–2.011	0.449	0.355–0.542

[a] Models controlled for age, gender (in the model for all), educational level, income, living arrangements, year and region

Supplementary table S6. Results of random (RE) and fixed effects (FE) regression analyses on the associations of work-related factors with part-time sickness absence (pSA) among men and women without mutual adjustment of work-related factors, percentage point differences

	pSA			
	RE[a]		FE[a]	
	**Estimate**	**95% CI**	**Estimate**	**95% CI**
**Men**				
Public sector (vs. private)	0.013	0.000–0.026	-0.128	-0.178– -0.078
Physically heavy work (20 percentage point increase in the % exposed)	0.011	0.007–0.015	0.005	-0.004–0.014
Job control (one unit decrease in the score)	0.123	0.109–0.137	0.038	0.017–0.059
**Women**				
Public sector (vs. private)	0.084	0.068–0.100	-0.188	-0.243– -0.132
Physically heavy work (20 percentage point increase in the % exposed)	0.100	0.091–0.108	-0.050	-0.068– -0.032
Job control (one unit decrease in the score)	0.153	0.133–0.173	0.088	0.048–0.127
**All**				
Public sector (vs. private)	0.067	0.056–0.078	-0.133	-0.174– -0.093
Physically heavy work (20 percentage point increase in the % exposed)	0.049	0.044–0.053	-0.010	-0.020– -0.001
Job control (one unit decrease in the score)	0.150	0.138–0.163	0.093	0.072–0.114

[a] Models controlled for age, gender (in the model for all), educational level, income, living arrangements, year and region

Supplementary table S7. Results of random (RE) regression analyses on the associations of work-related factors with full (fSA) and part-time sickness absence (pSA) among study subjects whose work-related factors changed over the study period, percentage point differences

	fSA		pSA	
	RE[a]		RE[a]	
	**Estimate**	**95% CI**	**Estimate**	**95% CI**
**Men**				
Public sector (vs. private)	0.602	0.338–0.866	-0.076	-0.121– -0.030
Physically heavy work (20 percentage point increase in the % exposed)	0.603	0.569–0.637	-0.004	-0.009–0.000
Job control (one unit decrease in the score)	2.035	1.938–2.132	0.076	0.061–0.092
**Women**				
Public sector (vs. private)	1.524	1.297–1.751	-0.096	-0.152– -0.040
Physically heavy work (20 percentage point increase in the % exposed)	1.416	1.365–1.466	0.042	0.031–0.053
Job control (one unit decrease in the score)	0.898	0.777–1.019	0.126	0.100–0.152
**All**				
Public sector (vs. private)	1.241	1.068–1.415	-0.077	-0.117– -0.037
Physically heavy work (20 percentage point increase in the % exposed)	0.916	0.887–0.944	0.011	0.006–0.016
Job control (one unit decrease in the score)	1.452	1.376–1.153	0.118	0.103–0.133

[a] Models controlled for age, gender, educational level, income, living arrangements, year, region and mutually for the work-related factors

Supplementary table S8. Results of random (RE) and fixed effects (FE) regression analyses on the associations of work-related factors with full sickness absence (fSA) among men and women using categorised occupational exposures, percentage point differences

	fSA			
	RE[a]		FE[a]	
	**Estimate**	**95% CI**	**Estimate**	**95% CI**
**Men**				
Public sector (vs. private)	3.379	3.275–3.484	-0.145	-0.427–0.138
Physically heavy work (% exposed; reference= ≤20)				
>20, ≤40	0.966	0.850–1.063	0.079	-0.062–0.221
>40, ≤60	2.041	1.936–0.022	0.264	0.118–0.411
>60	2.612	2.480–2.745	0.790	0.585–0.994
Job control (score quartiles; reference= highest control)				
2	1.300	1.217–1.376	0.018	-0.087–0.124
3	1.367	1.258–1.475	-0.206	-0.352– -0.060
4	3.171	3.070–3.272	0.699	0.567–0.831
**Women**				
Public sector (vs. private)	3.973	3.882–4.063	0.691	0.455–0.923
Physically heavy work (% exposed; reference= ≤20)				
>20, ≤40	3.003	2.900–3.105	0.298	0.131–0.465
>40, ≤60	4.572	4.435–4.709	0.870	0.650–1.090
>60	5.357	5.180–5.533	0.840	0.578–1.101
Job control (score quartiles; reference= highest control)				
2	1.141	1.030–1.251	0.094	-0.070–0.258
3	0.942	0.830–1.054	-0.037	-0.205–0.132
4	1.505	1.381–1.629	0.083	-0.104–0.268
**All**				
Public sector (vs. private)	3.845	3.778–3.913	0.386	0.202–0.568
Physically heavy work (% exposed; reference= ≤20)				
>20, ≤40	2.201	2.127–2.275	0.193	0.084–0.302
>40, ≤60	3.216	3.132–3.299	0.416	0.295–0.538
>60	3.872	3.767–3.978	0.749	0.587–0.911
Job control (score quartiles; reference= highest control)				
2	1.078	1.013–1.143	0.032	-0.058–0.122
3	1.062	0.987–1.137	-0.067	-0.173–0.039
4	2.222	2.145–2.300	0.376	0.268–0.483

[a] Models controlled for age, gender, educational level, income, living arrangements, year, region and mutually for the categorised work-related factors

Supplementary table S9. Results of random (RE) and fixed effects (FE) regression analyses on the associations of work-related factors with full sickness absence (pSA) among men and women using categorised occupational exposures, percentage point differences

	pSA			
	RE[a]		FE[a]	
	**Estimate**	**95% CI**	**Estimate**	**95% CI**
**Men**				
Public sector (vs. private)	0.034	0.021–0.047	-0.122	-0.172– -0.073
Physically heavy work (% exposed; reference= ≤20)				
>20, ≤40	0.037	0.021–0.054	0.027	0.000–0.054
>40, ≤60	0.022	0.007–0.038	-0.027	-0.054–0.000
>60	0.045	0.026–0.064	0.006	-0.030–0.043
Job control (score quartiles; reference= highest control)				
2	0.024	0.011–0.036	0.019	-0.002–0.040
3	0.008	-0.008–0.024	0.041	0.015–0.067
4	0.117	0.101–0.133	0.074	0.051–0.099
**Women**				
Public sector (vs. private)	0.107	0.090–0.123	-0.183	-0.238– -0.127
Physically heavy work (% exposed; reference= ≤20)				
>20, ≤40	0.105	0.080–0.130	0.012	-0.032–0.055
>40, ≤60	0.178	0.150–0.205	-0.028	-0.084–0.028
>60	0.257	0.220–0.294	-0.241	-0.321– -0.171
Job control (score quartiles; reference= highest control)				
2	0.160	0.137–0.183	0.008	-0.035–0.051
3	0.171	0.148–0.194	0.071	0.028–0.115
4	0.205	0.180–0.230	0.085	0.036–0.013
**All**				
Public sector (vs. private)	0.088	0.077–0.100	-0.130	-0.170– -0.089
Physically heavy work (% exposed; reference= ≤20)				
>20, ≤40	0.098	0.083–0.113	0.053	0.029–0.078
>40, ≤60	0.109	0.094–0.124	0.027	0.001–0.053
>60	0.127	0.108–0.146	-0.100	-0.137– -0.062
Job control (score quartiles; reference= highest control)				
2	0.071	0.058–0.084	0.032	0.011–0.053
3	0.075	0.061–0.089	0.078	0.054–0.102
4	0.153	0.139–0.168	0.104	0.080–0.129

[a] Models controlled for age, gender, educational level, income, living arrangements, year, region and mutually for the categorised work-related factors
